# High Complement Factor H-Related (FHR)-3 Levels Are Associated With the Atypical Hemolytic-Uremic Syndrome-Risk Allele CFHR3*B

**DOI:** 10.3389/fimmu.2018.00848

**Published:** 2018-04-24

**Authors:** Richard B. Pouw, Irene Gómez Delgado, Alberto López Lera, Santiago Rodríguez de Córdoba, Diana Wouters, Taco W. Kuijpers, Pilar Sánchez-Corral

**Affiliations:** ^1^Department of Immunopathology, Sanquin Research and Landsteiner Laboratory of the Academic Medical Center, University of Amsterdam, Amsterdam, Netherlands; ^2^Department of Pediatric Hematology, Immunology and Infectious Diseases, Emma Children’s Hospital, Academic Medical Center, Amsterdam, Netherlands; ^3^Complement Research Group, Hospital La Paz Institute for Health Research (IdiPAZ), La Paz University Hospital, Center for Biomedical Network Research on Rare Diseases (CIBERER), Madrid, Spain; ^4^Immunology Unit, Hospital La Paz Institute for Health Research (IdiPAZ), La Paz University Hospital, Center for Biomedical Network Research on Rare Diseases (CIBERER), Madrid, Spain; ^5^Biological Research Center (CIB)-CSIC, Center for Biomedical Network Research on Rare Diseases (CIBERER), Madrid, Spain; ^6^Department of Blood Cell Research, Sanquin Research and Landsteiner Laboratory of the Academic Medical Center, University of Amsterdam, Amsterdam, Netherlands

**Keywords:** complement, factor H, factor H-related protein 3, *CFHR3* gene, atypical hemolytic-uremic syndrome

## Abstract

Dysregulation of the complement alternative pathway (AP) is a major pathogenic mechanism in atypical hemolytic-uremic syndrome (aHUS). Genetic or acquired defects in factor H (FH), the main AP regulator, are major aHUS drivers that associate with a poor prognosis. FH activity has been suggested to be downregulated by homologous FH-related (FHR) proteins, including FHR-3 and FHR-1. Hence, their relative levels in plasma could be disease-relevant. The genes coding for FH, FHR-3, and FHR-1 (*CFH, CFHR3*, and *CFHR1*, respectively) are polymorphic and located adjacent to each other on human chromosome 1q31.3. We have previously shown that haplotype CFH(H3)–CFHR3*B–CFHR1*B associates with aHUS and reduced FH levels. In this study, we used a specific enzyme-linked immunosorbent assay to quantify FHR-3 in plasma samples from controls and patients with aHUS genotyped for the three known *CFHR3* alleles (CFHR3*A, CFHR3*B, and CFHR3*Del). In the 218 patients carrying at least one copy of *CFHR3*, significant differences between *CFHR3* genotype groups were found, with CFHR3*A/Del patients having the lowest FHR-3 concentration (0.684–1.032 µg/mL), CFHR3*B/Del and CFHR3*A/A patients presenting intermediate levels (1.437–2.201 µg/mL), and CFHR3*A/B and CFHR3*B/B patients showing the highest concentration (2.330–4.056 µg/mL) (*p* < 0.001). These data indicate that CFHR3*A is a low-expression allele, whereas CFHR3*B, associated with increased risk of aHUS, is a high-expression allele. Our study reveals that the aHUS-risk haplotype CFH(H3)–CFHR3*B–CFHR1*B generates twofold more FHR-3 than the non-risk CFH(H1)–CFHR3*A–CFHR1*A haplotype. In addition, FHR-3 levels were higher in patients with aHUS than in control individuals with the same *CFHR3* genotype. These data suggest that increased plasma levels of FHR-3, altering the balance between FH and FHR-3, likely impact the FH regulatory functions and contribute to the development of aHUS.

## Introduction

Atypical hemolytic-uremic syndrome (aHUS) is a thrombotic microangiopathy characterized by hematological and renal alterations, although neurological and cardiovascular damage is also frequent (1, 2). Genetic and/or acquired defects in the complement alternative pathway that disturb the activation-regulation balance are present in 40–60% of patients, potentiating the initial endothelial damage in the microvasculature (3–5). The prognosis of patients with aHUS who have mutations in complement factor H (FH) is particularly poor and is associated with terminal renal insufficiency at disease onset and disease recurrence in the transplanted kidney (6).

Factor H is the main complement regulator in the fluid phase, and it also binds to autologous cellular surfaces to protect them from complement-mediated damage (7). The FH gene, *CFH*, is located within a gene cluster that includes five additional genes (*CFHR1* to *CFHR5*) coding for the homologous FH-related proteins (FHR-1 to FHR-5), which likely compete with FH for ligand binding and act as complement deregulators (8, 9). Several aHUS-predisposing genetic variants within the *CFH/CFHR* gene cluster have been found. A particular *CFH* haplotype named *CFH(H3)* increases aHUS penetrance in carriers and modulates the clinical phenotype (10–12), and the homozygous deletion of the *CFHR3* and *CFHR1* genes (*Δ_CFHR3–CFHR1_*) predisposes patients to an autoimmune form of aHUS characterized by the generation of anti-FH blocking antibodies (13–15). Additional aHUS-risk variants within the *CFH/CFHR* region are the CFHR1*B and the CFHR3*B alleles (16, 17).

The molecular basis for the contribution of the genetic variants CFH(H3), CFHR3*B, and CFHR1*B to the pathogenic mechanism of aHUS have not yet been determined. These variants include non-synonymous single-nucleotide polymorphisms (SNPs) with potential functional consequences, but changes in gene expression cannot be excluded, particularly in the case of *CFH(H3)* and CFHR3*B, which include SNPs within their 5′-untranslated region (UTR).

We have shown that the aHUS-risk haplotype *CFH(H3)* is nearly always associated with the CFHR3*B and CFHR1*B alleles, thus generating an extended CFH(H3)–CFHR3*B–CFHR1*B haplotype, which predisposes to aHUS and favors a poorer progression of renal function at disease onset (17); we also demonstrated that patients homozygous for this haplotype have lower FH levels. To check the hypothesis that the aHUS-risk CFHR3*B allele (tagged by rs385390A; rs446868C; rs138675433C; rs149352569A) gives rise to higher protein levels than the non-risk CFHR3*A allele (rs385390C; rs446868A; rs138675433T; rs149352569T), we determined FHR-3 levels in patients with aHUS and in control individuals genotyped for CFHR3*A, CFHR3*B, and *Δ_CFHR3–CFHR1_* (referred to as CFHR3*Del). By using an FHR-3-specific enzyme-linked immunosorbent assay (ELISA) (18), we demonstrate that CFHR3*A is a low-expression allele and CFHR3*B is a high-expression allele and that increased FHR-3 levels in plasma are associated with aHUS.

## Materials and Methods

### Patients and Controls

A total of 230 patients from the Spanish aHUS registry with known *CFH, CFHR3*, and *CFHR1* genotypes were selected for the study. Genotyping had been previously determined by direct sequencing and copy number variation analyzed by multiplex ligation-dependent probe amplification (MLPA) or by using an in-house comparative genomic hybridization microarray (19). Studies to identify mutations in complement genes had also been performed on most of these patients. Blood samples were drawn during an acute aHUS episode or during remission, centrifuged to obtain serum and ethylenediaminetetraacetic acid (EDTA) plasma, and stored at −80°C until use. Peripheral-blood leukocytes (PBLs) were used to prepare genomic DNA by standard procedures. Plasma and DNA samples from 49 healthy Spanish individuals were also obtained and used in the study. Patients and controls provided written informed consent, as approved by the ethical committees from La Paz University Hospital or the Biological Research Center.

### *CFHR3* Genotyping

Genotyping of the CFHR3*A and CFHR3*B alleles was performed by Sanger sequencing of *CFHR3* exon 5, as described previously (17). The *CFHR3–CFHR1* genomic deletion was analyzed by using the SALSA MLPA probemix P236-A3 ARMD mix-1 (MRC-Holland, Amsterdam, Netherlands); this deletion is referred to as the CFHR3*Del allele, and it does not generate FHR-3 and FHR-1.

### FHR-3 Quantitation

FHR-3 levels in serum or EDTA plasma samples from the 230 patients with aHUS and the 49 controls were determined by using a specific sandwich FHR-3 ELISA as described previously (18).

### Statistical Analyses

The statistical significance of FHR-3 levels in the various geno-type or age groups was analyzed with IBM SPSS Software.

## Results

### FHR-3 Levels in Spanish Controls Suggest Differential Expression of CFHR3*A and CFHR3*B

**Figure 1:**
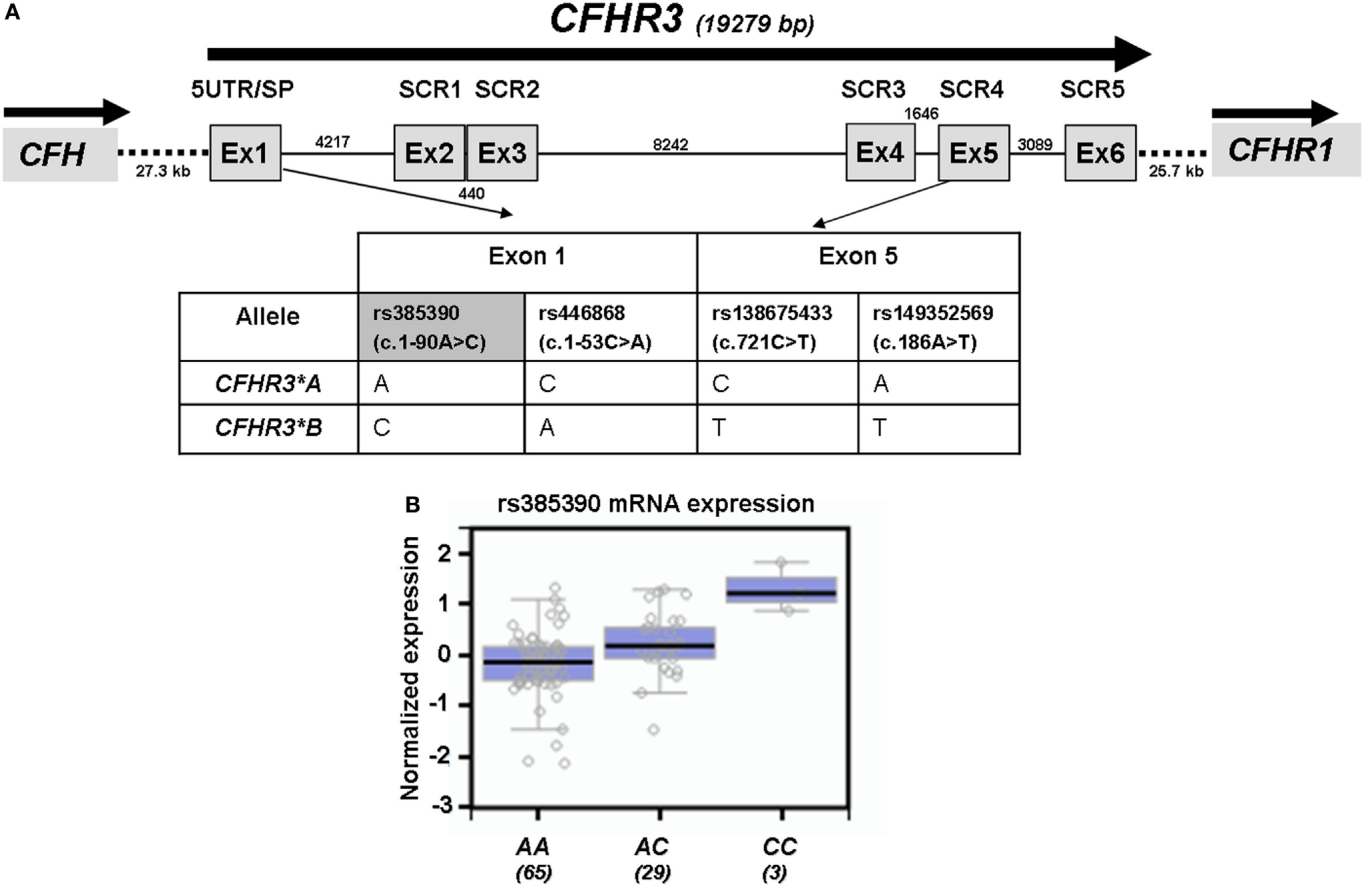
Genetic variants associated with the CFHR3*A and CFHR3*B alleles. **(A)** Exon–intron structure of the *CFHR3* gene showing the location of the genetic variants within exon 1 (rs385390; rs446868) and exon 5 (rs138675433; rs149352569) that tag the CFHR3*A and CFHR3*B alleles. **(B)** *In silico* data of gene expression correlations from the GTExPortal show that individuals who are homozygous for the rs385390 A variant present lower mRNA expression in liver tissue than individuals homozygous for the rs385390 C variant, or than heterozygous individuals. Number of individuals with each genotype is shown between brackets. GTExPortal: Portal for the Genotype-Tissue Expression project (https://www.gtexportal.org). GTExPortal is supported by the Common Fund of the National Institutes of Health. The data in panel **(B)** were obtained on October 2017.

We had previously determined FHR-3 levels in serum samples from 100 Dutch controls and had shown that FHR-3 concentration correlated with the number of *CFHR3* copies (18). We have now confirmed this observation in 47 Spanish control individuals containing 1 or 2 copies of *CFHR3*. FHR-3 levels ranged between 0.14 and 1.16 µg/mL (mean 0.61 ± 2.40; 95% CI 0.54–0.68), and FHR-3 concentration in individuals with 2 copies doubled the concentration observed in individuals with only one copy (0.68 vs. 0.36 µg/mL, *p* < 0.0001). Interestingly, genotyping of the Spanish controls for the CFHR3*A and CFHR3*B alleles provided additional data. FHR-3 levels were significantly lower in individuals with the CFHR3*A/A genotype (0.55 ± 0.15 µg/mL) than in individuals with the CFHR3*A/B (0.78 ± 0.18 µg/mL; *p* = 0.001) or CFHR3*B/B (0.82 ± 0.08 µg/mL; *p* = 0.033) genotypes, thus suggesting a lower expression of the CFHR3*A allele. A plausible explanation for this finding is that genetic variants located within the 5′-UTR of *CFHR3* exon 1 (rs385390 and rs446868) determine a different expression of the CFHR3*A and CFHR3*B alleles (Figure 1A). No data on the effect of these SNPs on *CFHR3* expression could be obtained from the Gene Expression Omnibus and Human Protein Atlas databases. However, data on gene expression correlations for the *CFHR3* SNP rs385390 (c.1-90A>C) available at the GTExPortal showed lower mRNA levels of the A variant (tagging the CFHR3*A allele) than the C variant (tagging the CFHR3*B allele) in liver tissue (Figure 1B).

### CFHR3*A Is a Low-Expression Allele, and CFHR3*B Is a High-Expression Allele

**Figure 2:**
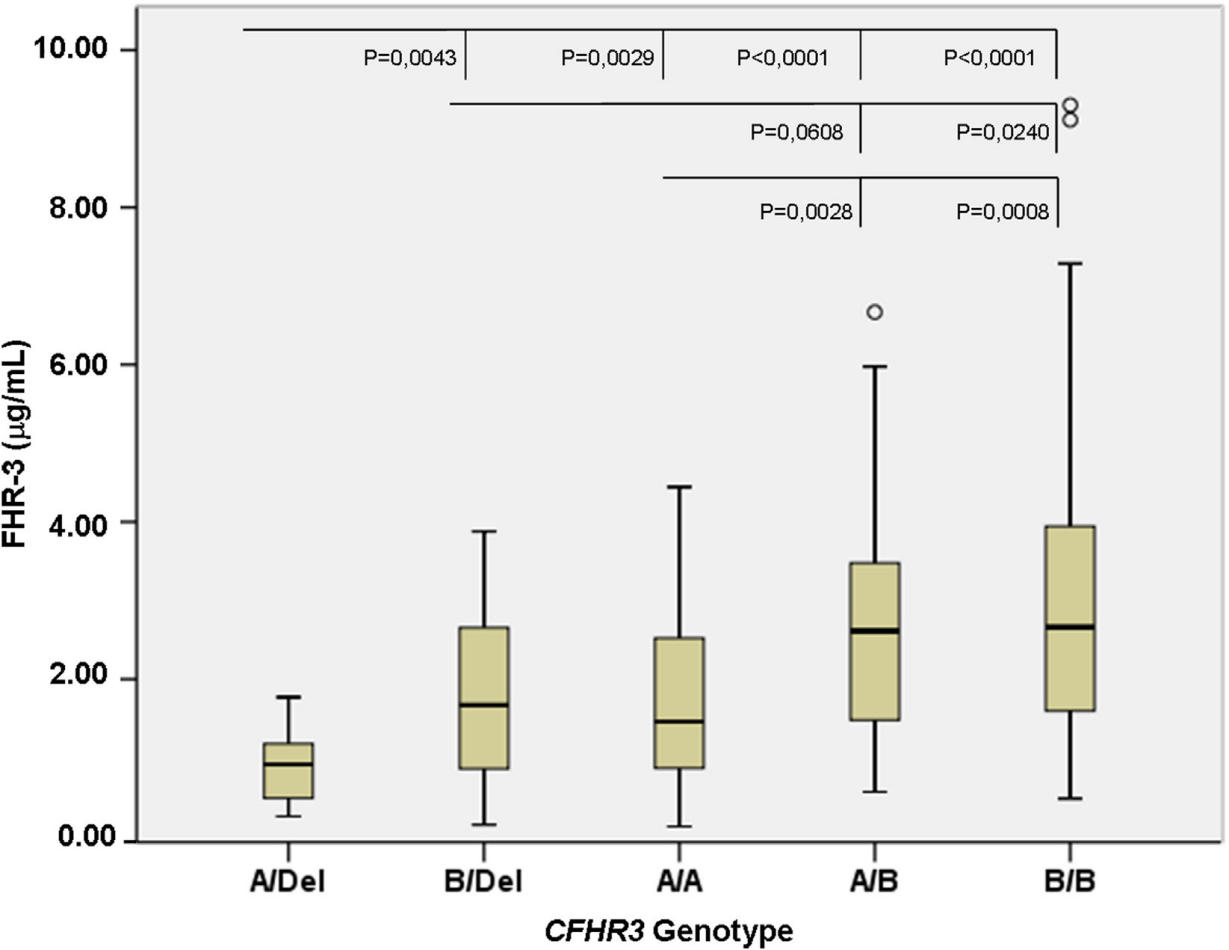
FHR-3 levels are associated with the CFHR3*A/B/Del genotypes. Boxplot of FHR-3 levels in 208 patients with atypical hemolytic uremic syndrome who were grouped according to their *CFHR3* genotype; patients with the *CFHR3 Del/Del* genotype were not included because they have homozygous FHR-3 deficiency. The statistical significance obtained in pairwise two-sided multiple comparisons is also shown. Outliers in the *CFHR3 A/B* and *B/B* genotype groups are denoted by symbols (o).

To confirm the association between FHR-3 levels and the CFHR3*A and CFHR3*B alleles, we determined FHR-3 levels in 230 patients with aHUS of known *CFHR3* genotypes (17). The 22 patients with aHUS with the CFHR3*Del/Del genotype presented minimal FHR-3 levels corresponding to the lower limit of detection of the ELISA. The other 208 patients, presenting *CFHR3* genotypes *A/Del, B/Del, A/A, A/B*, or *B/B*, showed a vast range of FHR-3 concentrations (0.124–13.450 µg/mL; mean 2.278 ± 1.723) (Table 1). Statistical analyses of these data were performed with nonparametric tests. The Kruskal–Wallis *H* test showed that there was a statistically significant difference in FHR-3 levels between the five *CFHR3* genotypes [χ^2^(4) = 53.568; *p* < 0.001]. Next, a pairwise two-sided multiple comparison analysis was performed using the Dwass-Steel-Critchlow-Fligner method. Significant differences between the *A/Del* and *B/Del* genotypes (*p* = 0.0043), as well as between the *A/A* and *B/B* genotypes (*p* = 0.0008) were found, thus revealing that the CFHR3*A allele was associated with lower FHR-3 concentrations than the CFHR3*B allele (Table 2; Figure 2).

**Table 1:** FHR-3 levels differ between *CFHR3* genotype groups.

Genotype	*N*	Mean	SD	SE	95% Confidence interval for mean		Minimum	Maximum
					Lower bound	Upper bound		
A/Del	24	0.85783	0.411379	0.083972	0.68412	1.03154	0.254	1.771
B/Del	32	1.82991	1.028643	0.182840	1.45904	2.20077	0.147	3.879
A/A	47	1.74011	1.033595	0.150765	1.43663	2.04358	0.124	4.443
A/B	58	2.70117	1.410761	0.185242	2.33023	3.07211	0.566	6.666
B/B	47	3.32538	2.487197	0.362795	2.59511	4.05565	0.481	13.450
Total	208	2.27832	1.722567	0.119438	2.04285	2.51379	0.124	13.450

*SPSS Descriptives table from the analysis of FHR-3 levels (µg/mL) in 208 patients with atypical hemolytic uremic syndrome grouped by *CFHR3* genotype. Statistically significant differences between the five *CFHR3* genotype groups were found (Kruskal–Wallis test, *p* < 0.0001)*.

**Table 2:** The CFHR3*A allele generates lower FHR-3 levels than the CFHR3*B allele.

Genotype	Wilcoxon Z	DSCF value	Pr > DSCF
A/Del vs. B/Del	−3.4935	4.9405	0.0043
A/Del vs. A/A	−3.6041	5.0970	0.0029
A/Del vs. A/B	−5.7174	8.0856	<0.0001
A/Del vs. B/B	−5.9927	8.4749	<0.0001
B/Del vs. A/A	0.4694	0.6638	0.9901
B/Del vs. A/B	−2.6552	3.7550	0.0608
B/Del vs. B/B	−2.9811	4.2159	0.0240
A/A vs. A/B	−3.6088	5.1036	0.0028
A/A vs. B/B	−3.9435	5.5770	0.0008
A/B vs. B/B	−0.8668	1.2258	0.9091

*Results from pairwise two-sided multiple comparisons done by Dwass-Steel-Critchlow-Fligner method. Pr > DSCF values lower than 0.05 denote statistically significant differences*.

### FHR-3 Levels and Genetic Background

Statistical analyses showed that plasma FHR-3 levels were associated with the CFHR3*A/B/del genotypes; however, protein levels within each *CFHR3* genotype group showed great variation. To explore the possibility that *CFHR3* gene expression was also modulated by genetic factors in the adjacent *CFH* and *CFHR1* genes, we analyzed the *CFH–CFHR3–CFHR1* genotypes of the 230 patients with aHUS. Genotyping of *CFH* (*H1/H2/H3/H4a/H4b/H5/H6/H7/H8*), *CFHR3* (*A/B/Del*), and *CFHR1* (*A/B/Del*) in these patients had already been performed [(17); Table S1 in Supplementary Material]. As many as 63 different *CFH–CFHR3–CFHR1* genotype combinations were observed (Table S2 in Supplementary Material), but only 23 of them were found in 3 or more patients (Table 3). The CFHR3*Del/Del genotype is associated with three *CFH* genotypic variants (*H4a, H4b*, or *H2)*, and 90% of patients with CFHR3*A/Del or CFHR3*B/Del present 1 out of 4 different *CFH–CFHR3–CFHR1* genotypes. Greater heterogeneity was found in the CFHR3*A/A and CFHR3*A/B groups, whereas 75% of the CFHR3*B/B patients were found to carry only two different *CFH-CFHR3-CFHR1* genotypes. However, when comparing FHR-3 levels within each *CFHR3* genotype group, no statistically significant differences between *CFH–CFHR3–CFHR1* genotypes were found, suggesting that genetic variants in *CFH* and *CFHR1* do not substantially contribute to the expression of the CFHR3*A and CFHR3*B alleles.

**Table 3:** *CFH–CFHR3–CFHR1* genotypes and FHR-3 levels.

	*CFH–CFHR3–CFHR1*genotype^a^			FHR-3 (µg/mL)		Number of patients	Frequency^b^ (%)
	*CFH*	*CFHR3*	*CFHR1*	Mean	SD		
G1 (22)	H4a,H4b	Del/Del	Del/Del	0.011	0.003	9	40.9
	H4a,H4a	Del/Del	Del/Del	0.010	0.000	7	31.8
	H2,H4a	Del/Del	Del/Del	0.022	0.024	4	18.2

G2 (24)	H2,H4a	A/Del	B/Del	0.784	0.363	7	29.2
	H1,H4b	A/Del	A/Del	0.778	0.444	6	25.0
	H1,H4a	A/Del	A/Del	0.719	0.418	5	20.8

G3 (32)	H3,H4a	B/Del	B/Del	2.155	0.862	15	46.9
	H3,H4b	B/Del	B/Del	1.872	0.981	7	21.9
	H3,H2	B/Del	B/Del	0.871	1.066	4	12.5
	H3,H4a	B/Del	A/Del	2.702	0.346	3	9.4

G4 (47)	H1,H1	A/A	A/A	1.871	0.867	12	25.5
	H1,H2	A/A	A/B	1.584	0.907	11	23.4
	H1,H2	A/A	A/A	2.297	1.152	4	8.5
	H1,H5	A/A	A/A	2.360	0.988	3	6.4
	H2,H2	A/A	B/B	1.679	2.397	3	6.4
	H2,H7	A/A	A/B	1.152	0.516	3	6.4

G5 (58)	H1,H3	A/B	A/B	2.445	1.171	17	29.3
	H2,H3	A/B	B/B	2.310	1.218	12	20.7
	H2,H3	A/B	A/B	3.241	0.749	6	10.3
	H3,H4a	A/B	B/B	2.889	1.226	3	5,2
	H1,H8	A/B	A/B	3.337	3.003	3	5.2

G6 (47)	H3,H3	B/B	B/B	3.673	3.048	28	59.6
	H3,H8	B/B	B/B	3.547	0.909	7	14.9

CFH and CFHR1 variants observed in patients with atypical hemolytic uremic syndrome presenting one or two copies of the CFHR3*A/B/Del alleles. Genotypes are ordered according to their relative frequency within the respective CFHR3 genotype subgroup (G1 to G6). FHR-3 levels within each CFH–CFHR3–CFHR1 genotype subgroup are also shown.

*^a^Minor *CFH–CFHR3–CFHR1* genotypes (i.e., those observed in 1 or 2 patients) are not included in the table*.

*^b^Frequency of each *CFH–CFHR3-–CFHR1* genotype within the corresponding subgroup (G1–G6); the total number of patients is shown between brackets*.

From the data in Table 3, we can also conclude that the CFHR3*Del allele mostly presents in the *CFH(H4a)* and *CFH(H4b)* haplotypes, the CFHR3*A allele in the *CFH(H1)* and *CFH(H2)* haplotypes and the CFHR3*B allele in the *CFH(H3)* and *CFH(H8)* haplotypes. These conclusions were further supported by the inferred *CFH–CFHR3–CFHR1* haplotypes. Among the 208 patients expressing FHR-3 (groups G2–G6 in Table 3), 150 patients had *CFH–CFHR3–CFHR1* genotypes homozygous for at least two genes, thus allowing segregation of the corresponding *CFH–CFHR3–CFHR1* chromosomes. Some 25 different *CFH–CFHR3–CFHR1* haplotypes were observed, the most frequent being CFH(H3)–CFHR3*B–CFHR1*B (31.7%), *CFH*(H4a)–CFHR3*Del–CFHR1*Del(20%) and *CFH*(H1)–CFHR3*A–CFHR3*A (16.3%). An analysis of FHR-3 levels in patients homozygous or hemizygous for these haplotypes rev-ealed higher expression in those patients with the CFH(H3)–CFHR3*B–CFHR1*B than *CFH*(H1)–CFHR3*A–CFHR3*A haplotype (1.87 ± 1.36 µg/mL vs. 0.9 ± 0.43 µg/mL; Kruskal–Wallis test, *p* < 0.001).

FHR-3 levels were also higher in patients with mutations in the complement genes *CFH, CFI, MCP, C3*, or *CFB*, than in patients without mutations (2.52 ± 1.62 vs. 2.06 ± 1.44 µg/mL, *p* < 0.05). This difference is probably explained by the different frequency of the CFHR3*B allele in patients with mutations (79%) and without mutations (59%).

### FHR-3 Levels Are Higher in Patients With aHUS Than in Controls With the Same *CFHR3* Genotype

**Figure 3:**
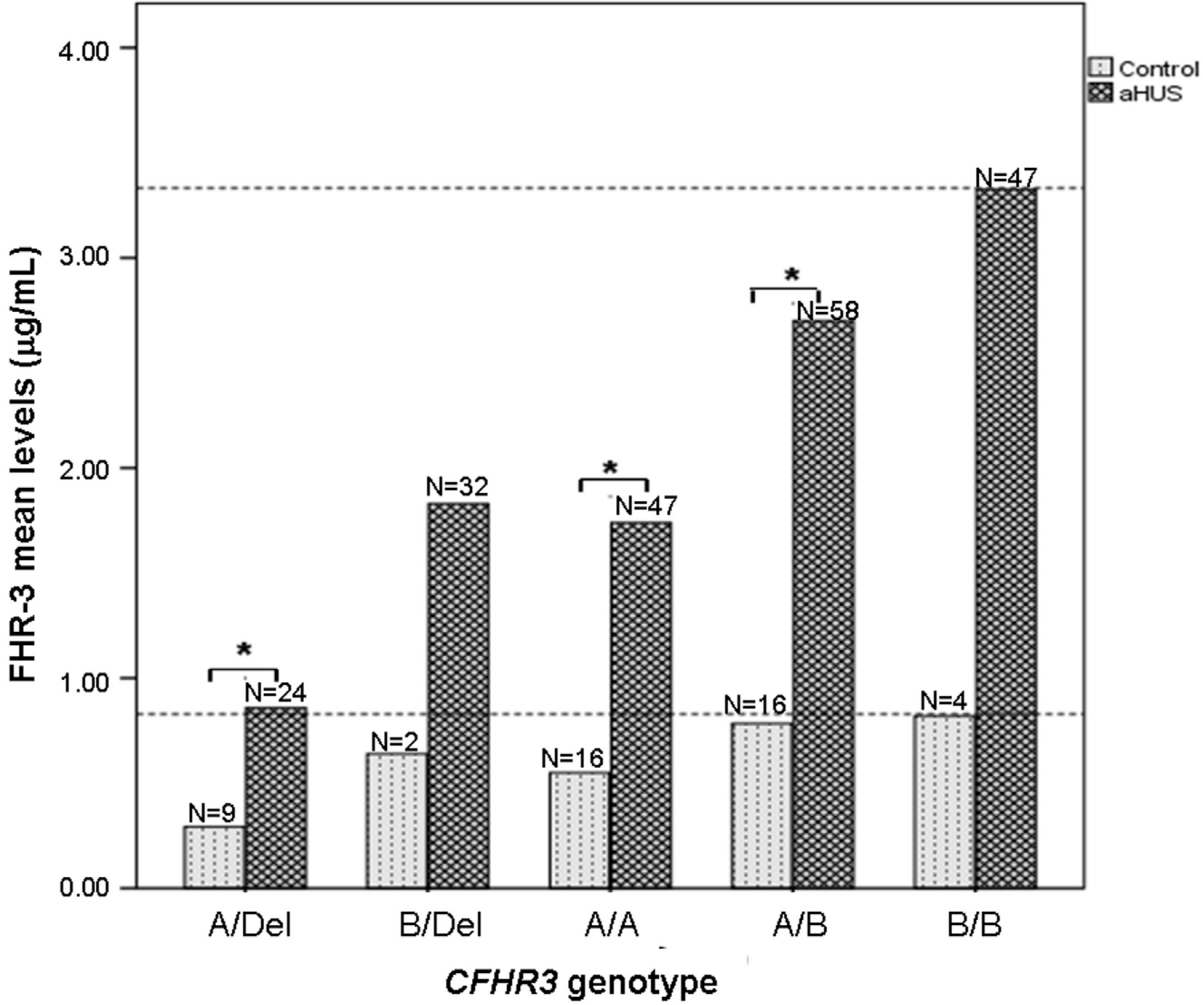
FHR-3 levels are higher in patients with atypical hemolytic uremic syndrome (aHUS) than in controls. Histogram showing the mean FHR-3 levels in controls and in patients with aHUS with different *CFHR3* genotypes. Statistically significant differences (Kruskal–Wallis test, *p* < 0.05) are denoted by an asterisk. Dashed lines indicate the highest FHR-3 level observed in controls and patients.

**Figure 4:**
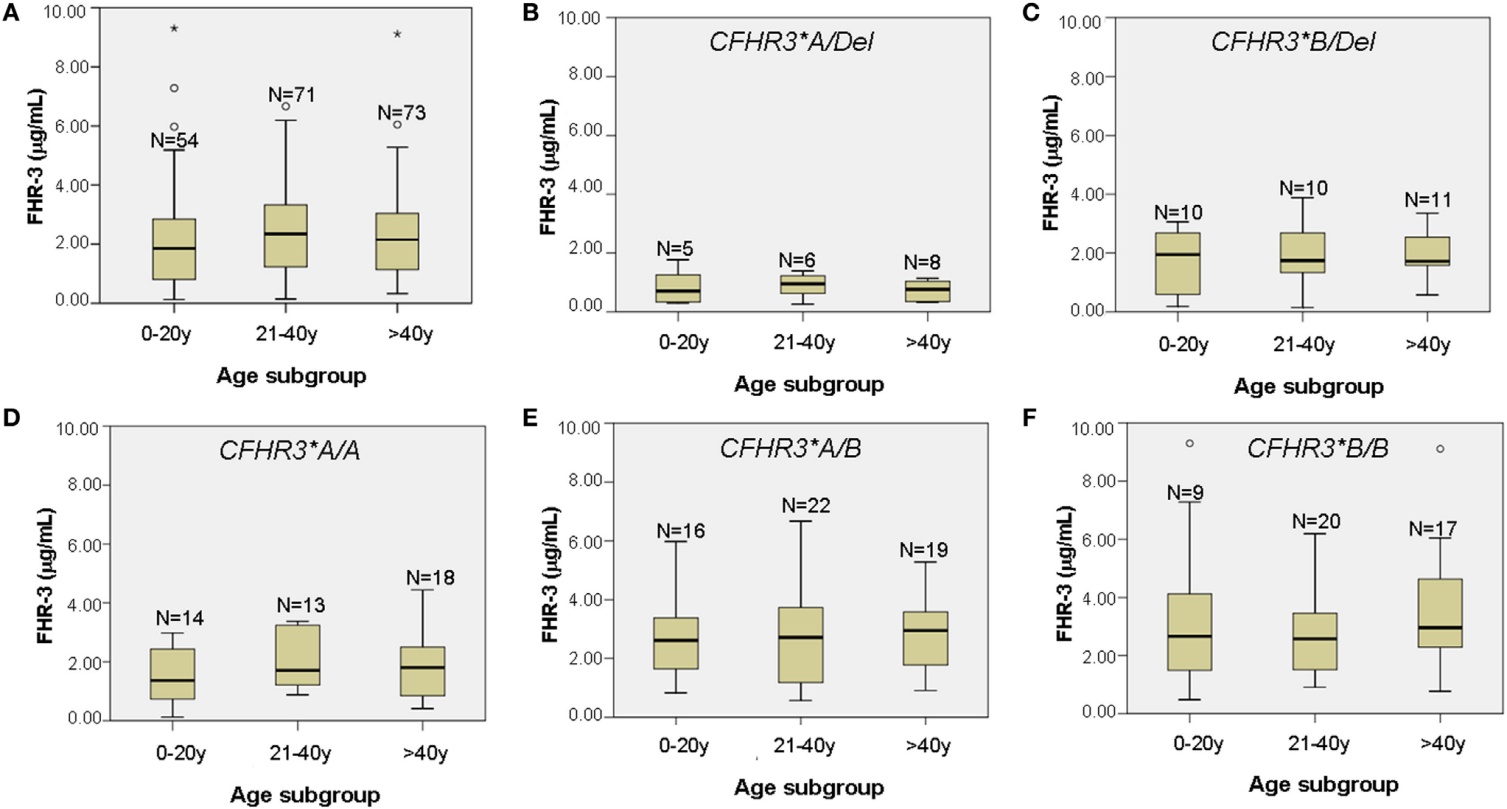
FHR-3 levels in patients with atypical hemolytic uremic syndrome (aHUS) do not change with age. **(A)** Boxplot of FHR-3 levels in 198 aHUS patients with the *CFHR3* genotypes *A/Del, B/Del, A/A, A/B*, or *B/B*, subgrouped according to their age at blood sampling (in years). **(B–F)** Boxplots of FHR-3 levels in each of the five *CFHR3* genotype groups. The number of patients within each age subgroup is also shown. Outliers are denoted by symbols (o).

Having observed that FHR-3 levels are associated with CFHR3*A/B/Del genotypes, we then compared FHR-3 levels in control individuals and in patients with aHUS with the same *CFHR3* genotype. FHR-3 levels were significantly higher in the patients with aHUS than in the controls with the same *CFHR3* genotypes *A/Del, A/A* or *A/B*, whereas in the *CFHR3* genotypes *B/Del* and *B/B* the differences in FHR-3 levels were not significant due to the small sample size of the control cohort (Figure 3). These results suggest that factors other than the CFHR3*A/B/Del alleles contribute to FHR-3 levels. To determine whether FHR-3 levels change with age, all patients with aHUS and at least one copy of *CFHR3* were included in one of three subgroups, according to their age at blood sampling. No significant differences in FHR-3 concentration were observed when comparing all the patients together, or when they were further subdivided according to their *CFHR3* genotype (Figure 4).

## Discussion

In this study, we show that the aHUS-risk CFHR3*B allele determines higher FHR-3 levels than the non-risk CFHR3*A allele. This observation was anticipated by the analysis of 49 healthy Spanish controls genotyped for the CFHR3*A/B/Del alleles and was further demonstrated in 230 patients with aHUS, most of Spanish origin.

FHR-3 levels in the 49 Spanish controls were 0.58 ± 0.26 µg/mL, which was very similar to the 0.69 µg/mL previously determined in 100 Dutch controls (18). In fact, these results support the evidence indicating that the actual concentration of FHR-3 in plasma is indeed much lower than the initial estimation of FHR-3 levels at 70–100 µg/mL (20). Moreover, we observed that the FHR-3 levels were significantly higher in the aHUS patients than in the control individuals (2.06 ± 1.77 vs. 0.58 ± 0.26 µg/mL, *p* < 0.0001). This result was not due to a significant difference in the allele frequency of CFHR3*Del between patients and controls, because when the 22 aHUS patients and the 2 controls with the CFHR3*Del/Del genotype and homozygous FHR-3 deficiency were excluded, a similar difference in FHR-3 levels was observed (2.28 ± 1.72 vs. 0.61 ± 0.24 µg/mL, *p* < 0.0001).

These results suggest that increased FHR-3 levels predispose to aHUS. This observation is in line with a previous study (21) that determined FHR-3 levels in 21 patients with aHUS (1.60 ± 0.57 µg/mL) and 21 controls (1.06 ± 0.53 µg/mL), although the difference in FHR-3 levels between their patient and control cohorts was smaller than in our study, which could well be related to the cohort size and the variation in allele frequency. In our study, 4% of the controls and 9% of the patients with aHUS carried the CFHR3*Del/Del genotype (i.e., homozygous *CFHR3-CFHR1* deletion), frequencies comparable to the 2.9% and 12.4% observed in a French study comparing 70 controls and 117 patients with aHUS (22). However, in the study by Schäfer et al. (Table S2 in Supplementary Material), as many as 14% of controls and 38% of patients with aHUS presented homozygous deficiency of FHR-3, and these high and very different frequencies limit the relevance of their observations when comparing aHUS patients and controls. High frequencies of homozygous *CFHR3–CFHR1* deletion have been reported in aHUS patients with anti-FH autoantibodies or with mutations in complement factor I (22), as well as in Middle Eastern and North African control populations (23, 24); the reason for the high frequency of the homozygous *CFHR3-CFHR1* deletion in certain control populations could be related with its protective role against age-related macular degeneration (25) and IgA nephropathy (26). In conclusion, the comparison of FHR-3 levels could be biased by the ethnic origin of controls and patients, and by the frequency of anti-FH autoantibodies or factor I mutations in the patient cohorts. These facts have to be taken into account when trying to establish proper comparisons between control and patient cohorts.

To adequately analyze the contribution of the CFHR3*A and CFHR3*B alleles to FHR-3 levels, patients and controls with the CFHR3*Del/Del genotype were excluded. An analysis of the control cohort suggested a higher expression of the CFHR3*B allele that was clearly confirmed in the aHUS cohort. Patients with the CFHR3*A/Del genotype showed the lowest FHR-3 levels (0.684–1.032 µg/mL), patients with genotypes *B/Del* and *A/A* presented intermediate levels (1.437–2.201 µg/mL), and patients with genotypes *A/B* and *B/B* had the highest FHR-3 levels (2.330–4.056 µg/mL), explaining the wide individual range in FHR-3 concentrations between 0.684 and 4.056 µg/mL.

More importantly, statistically significant differences in FHR-3 levels were observed between the *CFHR3* genotypes *A/Del* and *B/Del* (*p* = 0.0043), *A/A* and *A/B* (*p* = 0.0028), and *A/A* and *B/B* (*p* = 0.0008), demonstrating that the CFHR3*A allele is associated with lower FHR-3 concentrations than the aHUS-risk CFHR3*B allele. These data provide a genetic explanation for the increased FHR-3 levels observed in patients with aHUS, who present a higher frequency of the CFHR3*B allele than control individuals. However, whether higher FHR-3 levels increase susceptibility to aHUS is currently unknown, and the potential pathogenic mechanism will remain elusive until the actual physiological role of FHR-3 within the complement system is fully understood.

Recombinant FHR-3 binds C3b and C3d with similar affinity to FH (27); once bound, however, it cannot regulate complement activation because it lacks domains homologous to the N-terminal region of FH (28, 29). Therefore, increased competition between FHR-3 and FH for C3b binding will theoretically result in reduced complement regulation. Because the molar FHR-3 concentration in plasma is approximately 140 times lower than the molar FH concentration (18), competition between FHR-3 and FH for plasma-C3b binding is likely to be irrelevant. Nevertheless, the FHR-3/FH ratio could be more relevant for appropriate regulation of complement activation on cellular surfaces, such as the damaged endothelium of patients with aHUS. A recent study (30) shows a 1.3–1.9 increase in the FHR-1/FH molar ratio in patients with IgA nephropathy with disease progression. In our study, FHR-3 levels are 3.6 times higher in aHUS than in controls, while FH levels are only 1.2 times higher (not shown), but to demonstrate whether the increased FHR-3/FH ratio actually reduces complement regulation on the endothelial surface will require further investigation. Indirect evidence for competition between FH and FHR-3 on the cellular surface is provided by the functional characterization of a FH:FHR-3 hybrid protein identified in a familial case of aHUS (31). The authors suggest that the loss of FH regulatory activity of the hybrid FH:FHR-3 protein on cell-based assays could be explained because its C-terminal domains (belonging to FHR-3) prevent the correct orientation and function of the N-terminal domains (belonging to FH). A slightly different FH:FHR-3 hybrid protein, which also shows reduced FH regulatory activity on the cellular surface, has been reported in a sporadic case of aHUS (32). Based on these findings, we speculate that inefficient complement regulation on the cellular surface could also result from an altered FH/FHR-3 ratio. This would explain why the CFH(H3)–CFHR3*B–CFHR1*B haplotype, associated with reduced FH levels (17) and enhanced FHR-3 levels (this study) increases the risk of aHUS. The CFH(H3)-CFHR3*B–CFHR1*B haplotype also carries the *CFH* (rs1065489; p936D<E) and *CFHR3* variants (rs385390, rs426736, and rs371075) that were shown to confer protection against meningococcal disease (33); quantitation of FHR-3 levels and CFHR3*A/B/Del genotyping in these patients will likely help establish the actual relationship between this haplotype and protection against *N. meningitidis* infection.

Another interesting conclusion from our study is that FHR-3 concentration is not only determined by the number of copies of the CFHR3*A and CFHR3*B alleles. Stratification by the CFHR3*A/B/Del genotype still showed higher FHR-3 levels in patients with aHUS than in controls (Figure 3), suggesting that additional genetic and/or acquired factors contribute to the increased FHR-3 concentration in patients with aHUS. We cannot rule out the possibility that genetic variants within the adjacent *CFH* and *CFHR1* genes modulate *CFHR3* expression, but our current results do not favor this hypothesis (Table 3). Because plasma levels of FH have a wide range of variation and were shown to increase with age (34), we explored whether this was also the case for FHR-3 levels by analyzing data from the patients with aHUS. FHR-3 levels remain unchanged in the three age subgroups, either when considering the whole patient cohort, or when each *CFHR3* genotype was analyzed separately (Figure 4). A similar result was observed in the two age subgroups from the control cohort, but the small sample size and the absence of pediatric controls limit the relevance of this observation. Although FHR-3 does not appear to be an acute phase reactant (18), FHR-3 levels in patient samples could be associated with disease activity, in particular with the decreased renal function observed in aHUS. In this context, two recent studies in IgA nephropathy patients suggest that impaired renal function increases the FHR-1/FH ratio (30, 35). To fully understand the increased FHR-3 levels observed in patients with aHUS, it would be necessary to monitor FHR-3 concentration in serial samples from patients along their clinical evolution, and renal function/damage over time.

In conclusion, in this study we show that CFHR3*A is a low-expression allele, and CFHR3*B is a high-expression allele, and that, next to *CFHR3* copies, other genetic factors determine the FHR-3 levels. We also show that the aHUS-risk CFH(H3)–CFHR3*B–CFHR1*B haplotype is associated with increased FHR-3 levels, and speculate that it leads to an imbalance between the local FH and FHR-3 concentration that predisposes patients to aHUS. These results uncover that genotyping for the CFHR3*A, CFHR3*B, and CFHR3*Del alleles is necessary for a proper interpretation of FHR-3 levels within a certain pathological context, and we propose to incorporate these analysis to the current genetic workflow in aHUS patients.

## Ethics Statement

Patients and controls provided written informed consent, as approved by the ethical committees from La Paz University Hospital or the Biological Research Center.

## Author Contributions

RP, DW, and TK were responsible for quantitation of FHR-3 in patients and controls. IG genotyped the control individuals, performed statistical analyses, and prepared figures and tables. AL searched databases for *CFHR3* expression and prepared figures. SR was responsible for *CFH–CFHR3–CFHR1* genotyping in the patients with aHUS. PS-C designed the study, analyzed the data, and wrote the first draft of the manuscript. All the authors revised the data and contributed to the final version of the manuscript.

## Conflict of Interest Statement

The authors declare that the research was conducted in the absence of any commercial or financial relationships that could be construed as a potential conflict of interest.
