# Corrigendum: High Complement Factor H-Related (FHR)-3 Levels Are Associated With the Atypical Hemolytic-Uremic Syndrome-Risk Allele CFHR3^*^B

**DOI:** 10.3389/fimmu.2019.03073

**Published:** 2020-01-24

**Authors:** Richard B. Pouw, Irene Gómez Delgado, Alberto López Lera, Santiago Rodríguez de Córdoba, Diana Wouters, Taco W. Kuijpers, Pilar Sánchez-Corral

**Affiliations:** ^1^Department of Immunopathology, Sanquin Research and Landsteiner Laboratory of the Academic Medical Center, University of Amsterdam, Amsterdam, Netherlands; ^2^Department of Pediatric Hematology, Immunology and Infectious Diseases, Emma Children's Hospital, Academic Medical Center, Amsterdam, Netherlands; ^3^Complement Research Group, Hospital La Paz Institute for Health Research (IdiPAZ), La Paz University Hospital, Center for Biomedical Network Research on Rare Diseases (CIBERER), Madrid, Spain; ^4^Immunology Unit, Hospital La Paz Institute for Health Research (IdiPAZ), La Paz University Hospital, Center for Biomedical Network Research on Rare Diseases (CIBERER), Madrid, Spain; ^5^Biological Research Center (CIB)-CSIC, Center for Biomedical Network Research on Rare Diseases (CIBERER), Madrid, Spain; ^6^Department of Blood Cell Research, Sanquin Research and Landsteiner Laboratory of the Academic Medical Center, University of Amsterdam, Amsterdam, Netherlands

**Keywords:** complement, factor H, factor H-related protein 3, *CFHR3* gene, atypical hemolytic-uremic syndrome

There is an error in the **Acknowledgments** statement. The correct name for the funder “Spanish Ministerio de Economía y Competitividad” is “Spanish Instituto de Salud Carlos III.”

A correction has been made to the **Acknowledgments** section:

“We appreciate the technical assistance of Lorena Risueño, César Vélez, and Álex Otero in the management of biological samples of patients and controls. We thank Rosario Madero (IdiPAZ Biostatistics) for statistical help, and Vega Mauleón and Hoi Tong (IdiPAZ UCICEC) for blood sampling of the healthy Spanish volunteers. This study was funded by the Spanish Instituto de Salud Carlos III and the European Program FEDER (grants PI12/00597 and PI16/00723 to PS-C, and grant SAF2015-66287-R to SR), and by the Complement II-CM network (B2017/BMD-3673). RP and TK received funding from the European Union's seventh Framework program under EC-GA no. 279185 (EUCLIDS; www.euclids-project.eu). AL is supported by the Spanish Center for Biomedical Network Research on Rare Diseases (CIBERER).”

The authors apologize for this error and state that this does not change the scientific conclusions of the article in any way. The original article has been updated.

