# Supplementary material for: High Complement Factor H-Related (FHR)-3 Levels Are Associated With the Atypical Hemolytic-Uremic Syndrome-Risk Allele CFHR3*B
Source: Front Immunol. 2018 Apr 24;9:848. doi: 10.3389/fimmu.2018.00848 (PMC5928496; doi:10.3389/fimmu.2018.00848)
Supplement: Supplementary file 1 [file table_1.doc]

### Supplementary Table S1. Single nucleotide polymorphisms (SNPs) that define *CFH*, *CFHR3* and *CFHR1* haplotypes.

SNPs in *CFH*, *CFHR3* and *CFHR1* are named by their reference ID number (rs code) in the Human Genome Assembly GRCh38.p10 (Ensembl genome browser database). Nucleotides and amino acids are numbered according to the cDNA or protein notations of the Locus Reference Genome sequences LRG47 (*CFH* ENST00000367429.8 and [ENSP00000352658](http://www.ensembl.org/Homo_sapiens/Transcript/ProtVariations?db=core;source=dbSNP;t=ENSP00000352658.2;v=rs800292;vdb=variation;vf=571661).2), LRG175 (*CFHR3* [ENST00000367425](http://www.ensembl.org/Homo_sapiens/Transcript/Summary?db=core;source=dbSNP;t=ENST00000367425.8;v=rs138675433;vdb=variation;vf=24658424).8 and ENSP00000356395.4) and LRG149 (*CFHR1* ENST00000320493.9 and ENSP00000314299.5).

*this SNP is also known as IVS15-543G>A
