# Supplementary material for: High Complement Factor H-Related (FHR)-3 Levels Are Associated With the Atypical Hemolytic-Uremic Syndrome-Risk Allele CFHR3*B
Source: Front Immunol. 2018 Apr 24;9:848. doi: 10.3389/fimmu.2018.00848 (PMC5928496; doi:10.3389/fimmu.2018.00848)
Supplement: Supplementary file 2 [file table_2.doc]

### Supplementary Table S2. *CFH-CFHR3-CFHR1* genotypes in 230 patients with aHUS.

Genotypes are listed according to their relative frequency.

| ***CFH*** | ***CFHR3*** | ***CFHR1*** | **Number** | **Frequency (%)** |
| --- | --- | --- | --- | --- |
| H3,H3 | B/B | B/B | 28 | 12.17 |
| H1,H3 | A/B | A/B | 17 | 7.39 |
| H3,H4a | B/Del | B/Del | 15 | 6.52 |
| H1,H1 | A/A | A/A | 12 | 5.22 |
| H2,H3 | A/B | B/B | 12 | 5.22 |
| H1,H2 | A/A | A/B | 11 | 4.78 |
| H4a,H4b | Del/Del | Del/Del | 9 | 3.91 |
| H2,H4a | A/Del | B/Del | 7 | 3.04 |
| H3,H4b | B/Del | B/Del | 7 | 3.04 |
| H3,H8 | B/B | B/B | 7 | 3.04 |
| H4a,H4a | Del/Del | Del/Del | 7 | 3.04 |
| H1,H4b | A/Del | A/Del | 6 | 2.61 |
| H2,H3 | A/B | A/B | 6 | 2.61 |
| H1,H4a | A/Del | A/Del | 5 | 2.17 |
| H1,H2 | A/A | A/A | 4 | 1.74 |
| H2,H3 | B/Del | B/Del | 4 | 1.74 |
| H2,H4a | Del/Del | Del/Del | 4 | 1.74 |
| H1,H5 | A/A | A/A | 3 | 1.30 |
| H1,H8 | A/B | A/B | 3 | 1.30 |
| H2,H2 | A/A | B/B | 3 | 1.30 |
| H2,H7 | A/A | A/B | 3 | 1.30 |
| H3,H4a | A/B | B/B | 3 | 1.30 |
| H3,H4a | B/Del | A/Del | 3 | 1.30 |
| H1,H3 | A/A | A/A | 2 | 0.87 |
| H2,- | A/B | A/B | 2 | 0.87 |
| H2,H3 | B/B | B/B | 2 | 0.87 |
| H2,H5 | A/B | A/B | 2 | 0.87 |
| H3,- | B/B | A/A | 2 | 0.87 |
| H3,- | B/B | B/B | 2 | 0.87 |
| H3,H3 | A/A | B/B | 2 | 0.87 |
| H3,H3 | A/B | B/B | 2 | 0.87 |
| H3,H3 | B/B | A/B | 2 | 0.87 |
| H3,H5 | A/B | A/B | 2 | 0.87 |
| H4a,H4a | A/Del | B/Del | 2 | 0.87 |
| H1,- | A/A | A/A | 1 | 0.43 |
| H1,- | B/B | A/B | 1 | 0.43 |
| H1,H1 | A/A | A/DelR1R4 | 1 | 0.43 |
| H1,H2 | A/Del | A/Del | 1 | 0.43 |
| H1,H3 | A/B | A/A | 1 | 0.43 |
| H1,H4b | B/B | A/A | 1 | 0.43 |
| H1,H6 | A/A | A/A | 1 | 0.43 |
| H1,H7 | A/A | A/A | 1 | 0.43 |
| H2,- | B/Del | B/Del | 1 | 0.43 |
| H2,H3 | B/B | A/B | 1 | 0.43 |
| H2,H4a | B/Del | B/Del | 1 | 0.43 |
| H2,H4b | Del/Del | Del/Del | 1 | 0.43 |
| H2,H5 | A/A | A/B | 1 | 0.43 |
| H3,- | A/B | A/B | 1 | 0.43 |
| H3,- | A/B | B/B | 1 | 0.43 |
| H3,H3 | A/A | A/A | 1 | 0.43 |
| H3,H4a | A/B | A/B | 1 | 0.43 |
| H3,H4a | A/Del | B/Del | 1 | 0.43 |
| H3,H4b | A/B | A/A | 1 | 0.43 |
| H3,H4b | A/B | B/B | 1 | 0.43 |
| H3,H4b | B/B | B/B | 1 | 0.43 |
| H3,H7 | A/B | A/B | 1 | 0.43 |
| H3,H8 | A/B | B/B | 1 | 0.43 |
| H4a, - | Del/Del | Del/Del | 1 | 0.43 |
| H4a, H7 | A/A | A/B | 1 | 0.43 |
| H4a,H4b | A/Del | B/Del | 1 | 0.43 |
| H4a,H4b | B/Del | B/Del | 1 | 0.43 |
| H4b,H7 | A/Del | A/Del | 1 | 0.43 |
| H5,- | A/B | A/B | 1 | 0.43 |
